# *KLB* and *NOX4* expression levels as potential blood-based transcriptional biomarkers of physical activity in children

**DOI:** 10.1038/s41598-023-31537-4

**Published:** 2023-04-05

**Authors:** Sebastià Galmés, Azahara I. Rupérez, Juana Sánchez, Luis A. Moreno, Ronja Foraita, Antje Hebestreit, Dénes Molnár, Andreu Palou, Catalina Picó

**Affiliations:** 1 Laboratory of Molecular Biology, Nutrition and Biotechnology (Group of Nutrigenomics, Biomarkers and Risk Evaluation – NuBE), University of the Balearic Islands, Cra. Valldemossa Km 7.5, 07122 Palma, Spain; 2 Health Research Institute of the Balearic Islands (IdISBa), 07120 Palma, Spain; 3 Centro de Investigación Biomédica en Red de Fisiopatología de la Obesidad y Nutrición, Instituto de Salud Carlos III, 28029 Madrid, Spain; 4 GENUD (Growth, Exercise, Nutrition and Development) Research Group, Faculty of Health Sciences, University of Zaragoza, 50009 Zaragoza, Spain; 5 Instituto Agroalimentario de Aragón (IA2) and Instituto de Investigación Sanitaria de Aragón (IIS Aragón), Zaragoza, Spain; 6 Leibniz Institute for Prevention Research and Epidemiology—BIPS, 28359 Bremen, Germany; 7 Medical School and National Laboratories of Human Reproduction, University of Pécs, 7624 Pécs, Hungary

**Keywords:** Gene expression, Biomarkers

## Abstract

Insufficient physical activity (PA) in children is considered one of the major contributors to obesity and cardiometabolic complications later in life. Although regular exercise may contribute to disease prevention and health promotion, reliable early biomarkers are required to objectively discern people performing low PA from those who exercise enough. Here, we aimed to identify potential transcript-based biomarkers through the analysis of a whole-genome microarray in peripheral blood cells (PBC) from physically less active (n = 10) comparing with more active (n = 10) children. A set of genes differentially expressed (p < 0.01, Limma test) in less physically active children were identified, including the down-regulation of genes related to cardiometabolic benefits and improved skeletal function (*KLB*, *NOX4*, and *SYPL2*), and the up-regulation of genes whose elevated expression levels are associated with metabolic complications (*IRX5*, *UBD*, and *MGP*). The analysis of the enriched pathways significantly affected by PA levels were those associated with protein catabolism, skeletal morphogenesis, and wound healing, among others, which may suggest a differential impact of low PA on these processes. Microarray analysis comparing children according to their usual PA has revealed potential PBC transcript-based biomarkers that may be useful in early discerning children expending high sedentary time and its associated negative consequences.

## Introduction

Childhood obesity is a worrying problem worldwide, and it is associated with other comorbidities such as metabolic syndrome, insulin resistance/impaired glucose tolerance, and non-alcoholic fatty liver disease, among others^1^. It has been shown that sedentary lifestyle and lack of a sufficient time of physical activity (PA), established at a minimum of one hour per day of moderate to vigorous PA (MVPA), are associated with obesity^2^ and an overall poorer health-related quality of life^3^. Conversely, epidemiological studies have shown that regular PA is one of the most important contributors to disease prevention and health promotion, especially in children^4^. In this sense, the World Health Organization (WHO) has developed a global guideline to implement services and programs to increase PA and limit sedentary behavior in children, adolescents, adults, older adults, including population subsets with specific physiological conditions^5^, supporting that the practice of PA is associated with reduced risk of diseases progression (including type 2 diabetes, hypertension, cancer, etc.), premature mortality and improved life quality^6^. Focusing on children, evidence from a systematic review supports that both the time expended and the intensity of PA are associated with beneficial effects on health^7^. Following these lines, the interruption of sedentary associated-behavior in children is suggested as a promising strategy to reduce cardiometabolic abnormalities^8,9^. Therefore, moving away from sedentary behavior is key to improving the cardiometabolic health of the youth population, especially after the negative impact of the pandemic and home lockdown^10^. However, the classification of sedentary behaviors or low physical activity is largely based on methodologies that include self-report and subjective measurement^11^ or needs the usage of continuous reading instruments (i.e. accelerometers) and subsequent data interpretation^12^. In this context, there is a need to identify early novel candidate markers, or a combination of them, that may be applicable to children that allow to objectively discern individuals who are physically inactive or perform insufficient PA, which is critical to prevent sedentary behaviors more efficiently.

Gene expression analyses allow for the discovery of candidate genes and molecular mechanisms that may be involved in the development of diseases such as obesity and metabolic disturbances, among many others. In this sense, peripheral blood cells (PBC), or the subfraction of peripheral blood mononuclear cells (PBMC) that include lymphocytes and monocytes, are an easy-obtaining source for gene expression analyses^13,14^, since they can reflect gene expression patterns occurring in less accessible tissues, such as the adipose tissue or liver, and provide information on physiological and pathological conditions of the organism^15–18^. Previous studies conducted in the frame of the Identification and prevention of Dietary- and lifestyle-induced health EFfects In Children and infantS (IDEFICS) study showed a differential expression between children with or without obesity^19^. Regarding PA, previous studies have reported changes in gene expression in blood cells after acute exercise interventions in children and adolescents^20,21^, suggesting that these cells are responsive to PA. However, existing evidence in this field on the transcriptional response to sedentary behavior and PA in pediatric populations is rather limited, and more studies are required, as concluded in a recent systematic review^22^. In addition, the impact of regular PA habits or, conversely, the effects of low PA level or sedentary behavior on PBC gene expression pattern in children have not been directly evaluated. This may be useful to find out pathways and molecular mechanisms behind the beneficial/adverse effects of PA/sedentary lifestyle, respectively, and to identify objective candidate biomarkers of sedentary behavior.

Here, we aimed to identify the differential gene expression profile in PBC of children with lower PA level vs. those with higher level, according to data on daily MVPA collected by accelerometers and the cut-offs proposed by Evenson et al.^23^. To this end, we used the results of a whole-genome array analysis carried out in PBC from a subset of children from the Spanish cohort of the IDEFICS study and analysed the differentially expressed genes between subjects with lower MVPA levels versus those with higher levels. The results obtained are expected to allow the identification of candidate transcriptomic-based biomarkers associated with physical inactivity or a less physically active lifestyle that may be useful to address more targeted early interventions to prevent sedentary behavior and hence improve health.

## Methods

### Participants

The present study was carried out in a subgroup of the Spanish cohort of participants from the IDEFICS study, a large European multi-center study on childhood obesity (see study design in^24^), for whom we had whole-genome microarray expression data (32 children, including 16 males and 16 females)^19^. After excluding individuals with missing accelerometer data or near to zero values on MVPA, the sample size was 28 (14 males and 14 females). The study was approved by the Clinical Research Ethics Committee of Aragon (CEICA, code: 11/2007) and all procedures were followed in accordance with the Declaration of Helsinki.

### Objective evaluation of physical activity

**Figure 1:**
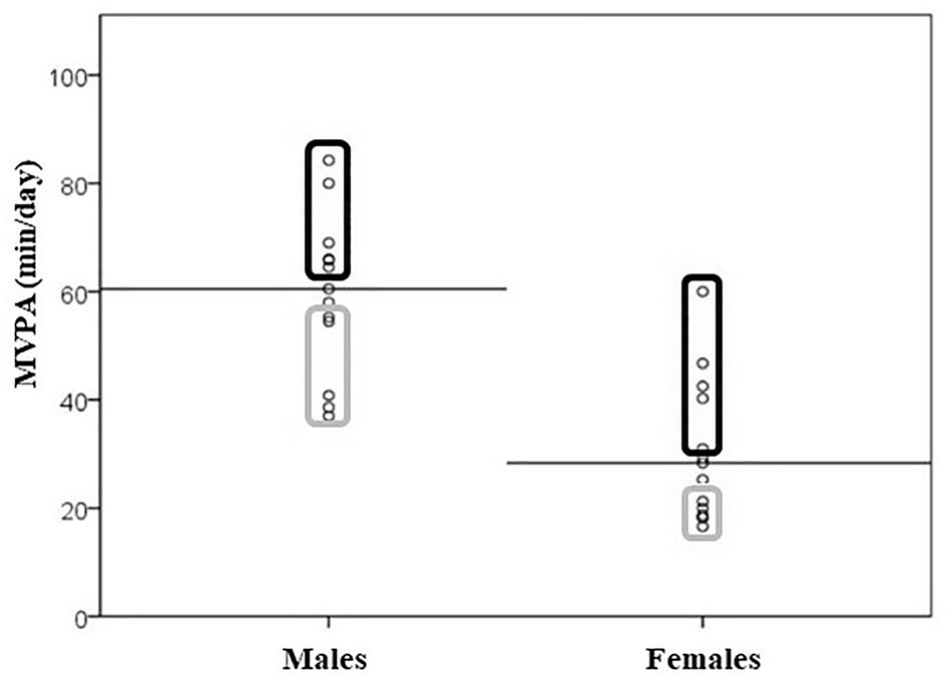
Moderate-vigorous physical activity (MVPA) duration (min/day) for males and females, categorized as active (black line box) or inactive (grey line box). Horizontal lines represent the median MVPA value for each sex. The MVPA values were obtained from child-worn accelerometer data for three consecutive days (including a weekend day), according Evanson's cut-offs.

We used previously anthropometry and blood biochemical markers that were measured as described^24^. MVPA was monitored by means of a uniaxial accelerometer (Actigraph or ActiTrainer, ActiGraph, Pensacola, USA) that children wore on a hip belt over three consecutive days, including one weekend day (considered enough time to accurately estimate MVPA in children^25–27^, taking it off for shower, water-based activities, and sleep. The device was set to record PA in 15 s epochs and counts per minute (cpm) were used to categorize intensities following the cut-offs proposed by Evenson: sedentary, 0–100 cpm; light, 101–2295 cpm; moderate, 2296–4011 cpm; and vigorous, ≥ 4012 cpm^23,28^. Raw minutes were divided by wearing time and multiplied by the average wearing time. Moderate and vigorous times were added to obtain daily MVPA times in minutes. Next, children were categorized according to their habitual MVPA level as less or more active if they had MVPA duration (min/day) above or below the median for their sex, respectively (Fig. 1). To enhance the statistical power of the comparison, subsequent analyses were performed with the 5 most and 5 least active boys and girls in terms of their MVPA, excluding 4 participants of both sexes with an MVPA value around the median, which resulted in a final number of 20 individuals.

### Gene expression analysis

We used previously acquired whole-genome microarray expression data (4 × 44 K G4845A human whole-genome Agilent microarray) in PBC when comparing normal weight and overweight individuals of the Spanish IDEFICS study^19^. A detailed description of the methodology can be found in^19^. In the present study, expression values in PBC were re-analysed by comparing more active and less active individuals based on their MVPA values according to the median value of their own sex. The array results were validated through quantitative polymerase chain reaction of 10 selected genes, as previously published^19^.

### Statistical analyses

Description of the study sample by sex was made as the mean and standard deviation of age, weight, height, body mass index (BMI), daily MVPA duration, total cholesterol (TC), high-density lipoprotein cholesterol (HDL-c), triacylglycerides (TG), low-density lipoprotein cholesterol (LDL-c), Non-HDL-cholesterol, TC:HDL-c ratio, glucose, insulin, homeostasis model assessment of insulin resistance (HOMA-IR), high-sensitivity C-reactive protein (hsCRP) and glycated hemoglobin (HbA1c). Statistical differences between groups (boys vs. girls and low vs. high MVPA) were assessed by U the Mann–Whitney test.

The differential gene expression between children with shorter and longer daily MVPA durations was assessed by the Limma test within the Babelomics 5 platform (http://babelomics.bioinfo.cipf.es)^29^ as previously described^19^. Adjusted multiple testing p-values using False Discovery Rate (FDR) were also obtained from this platform. The reference category used was the higher active group (longer daily duration in MVPA) in boys and girls. Fold change (FC) gene expression values between both groups were obtained by the difference of the mean of individual log2 values and these calculations were performed in Microsoft Excel (Microsoft 365, Washington, United States). Volcano plot of the microarray data was made using Microsoft Excel (Microsoft 365, Washington, United States) representing FC (log2) and Limma test p-values (− log10).

The threshold of significance for Limma test was set at p < 0.01 and genes whose p-value was below the established threshold were included for further analyses. These were manually classified into biological processes using available databases (Genecards and NCBI). Identified genes with a known function were plotted using the R based Heatmapper tool (http://www2.heatmapper.ca/expression/)^30^, using the centroid hierarchical linkage clustering method with Pearson distance. Moreover, sequences differently expressed were used to perform pathway enrichment analysis using MetaScape database software^31^ to classify these genes into biological pathways and establish the pathways that are most affected by the level of PA.

### Consent statement

Parental informed consent was obtained from all subjects involved in the study.

## Results

### Participants

The number of individuals included in the statistical analysis was 20 (including 10 girls and 10 boys). The individuals ranged between 4 and 8 years of age and general characteristics are shown in Table 1. Comparing between boys and girls, considerable differences were observed in relation to triacylglyceride levels and total cholesterol/HDL-cholesterol ratio, which were greater in females than in males, and, above all, PA level, with males showing almost double the daily MVPA duration (59 min/day) compared to females (31 min/day). Table 2 shows general characteristics of children, once categorized as more active and less active based on their daily MVPA duration according to the median value of their own sex. No differences were found between more active and less active children on studied anthropometric or biochemical markers except for daily MVPA duration, either when comparing both sexes together or analysed separately.

**Table 1:** Anthropometry, physical activity, and biochemical marker characteristics of the study participants.

	Males			Females			p-value
	N	Mean	SD	N	Mean	SD	
Age (years)	10	6.04	1.44	10	6.46	1.21	0.519
Weight (kg)	10	26.3	10.1	10	26.7	11.4	0.821
Height (cm)	10	115	12.1	10	120	12.2	0.364
BMI (kg/m^2^)	10	19.0	3.79	10	17.8	4.92	0.473
BMI Z-score	10	1.33	1.56	10	0.66	2.21	0.496
Hip circumference (cm)	10	66.7	12.2	10	67.1	13.1	0.880
FFMI	10	12.2	1.74	10	10.9	1.65	0.112
% Body fat	10	35.0	5.55	10	36.6	8.89	0.880
Waist/hip ratio	10	0.90	0.03	10	0.90	0.06	0.790
Waist-to-height ratio	10	0.52	0.06	10	0.50	0.09	0.450
MVPA (min/day)	**10**	**59.1**	**16.8**	**10**	**31.2**	**15.3**	**0.005**
Total cholesterol (mg/dL)	10	148	30.0	9	159	33.6	0.414
HDL-cholesterol (mg/dL)	10	55.5	13.5	9	46.4	8.63	0.110
LDL-cholesterol (mg/dL)	10	82.7	26.7	9	99.0	26.5	0.348
Non-HDL-cholesterol (mg/dL)	10	92.1	26.6	9	112	28.0	0.191
Total cholesterol/HDL	**10**	**2.76**	**0.63**	**9**	**3.44**	**0.59**	**0.034**
Triacylglycerides (mg/dL)	**10**	**47.2**	**6.96**	**9**	**67.1**	**27.2**	**0.029**
Glucose (mg/dL)	10	82.1	7.82	9	85.9	9.64	0.595
Insulin (µIU/mL)	8	5.61	3.04	8	6.45	5.27	0.916
HOMA-IR	8	1.18	0.68	8	1.36	1.05	0.834
HbA1c [%]	10	5.32	0.39	8	5.30	0.24	0.558
hsCRP (mg/dL)	9	0.21	0.25	8	0.25	0.29	0.664

Statistically significant differences were assessed *U* the Mann–Whitney test and are indicated in bold. *BMI* body mass index, BMI Z-score, according to Cole et al.^32^, *FFMI* Fat-free mass index, *MVPA* moderate-vigorous physical activity, *HDL-Cholesterol* high-density lipoprotein cholesterol, *LDL-Cholesterol* low-density lipoprotein cholesterol, *HOMA-IR* homeostasis model assessment for insulin resistance, *HbA1c* glycated hemoglobin, *hsCRP* high sensitivity C-reactive protein.

**Table 2:** Anthropometry, physical activity, and biochemical marker characteristics of the study participants according to their physical activity level and sex.

	Total sample							Males							Females						
	Low MVPA			High MVPA			p-value	Low MVPA			High MVPA			p-value	Low MVPA			High MVPA			p-value
	N	Mean	SD	N	Mean	SD		N	Mean	SD	N	Mean	SD		N	Mean	SD	N	Mean	SD	
Age (years)	10	6.41	1.33	10	6.09	1.35	0.569	5	6.62	1.41	5	5.46	1.36	0.248	5	6.20	1.37	5	6.72	1.12	0.598
Weight (kg)	10	26.5	10.0	10	26.5	11.4	0.940	5	28.8	9.55	5	23.8	11.1	0.465	5	24.2	11.1	5	29.1	12.3	0.465
Height (cm)	10	118	10.5	10	118	14.1	0.910	5	119	10.7	5	111	12.9	0.295	5	116	11.3	5	125	12.5	0.251
BMI (kg/m^2^)	10	18.6	4.67	10	18.2	4.18	0.910	5	19.6	3.43	5	18.4	4.43	0.754	5	17.6	5.90	5	18.0	4.42	0.465
BMI Z-score	10	1.04	2.11	10	0.95	1.76	0.940	5	1.64	1.26	5	1.02	1.92	0.754	5	0.44	2.75	5	0.88	1.82	0.754
Hip circumference (cm)	10	66.9	12.4	10	67.0	12.9	0.970	5	69.8	11.2	5	63.7	13.6	0.295	5	63.9	14.1	5	70.3	12.7	0.347
FFMI	10	11.7	1.79	10	11.5	1.86	0.762	5	12.7	1.31	5	11.8	2.15	0.602	5	10.7	1.78	5	11.1	1.70	0.754
% Body fat	10	35.4	8.60	10	36.1	6.09	0.650	5	34.6	5.86	5	35.3	5.89	0.754	5	36.2	11.4	5	36.9	6.86	0.754
Waist/hip ratio	10	0.91	0.05	10	0.89	0.04	0.704	5	0.89	0.03	5	0.91	0.03	0.461	5	0.92	0.06	5	0.87	0.05	0.172
Waist-to-height ratio	10	0.51	0.08	10	0.51	0.07	0.940	5	0.52	0.04	5	0.52	0.07	0.917	5	0.51	0.12	5	0.49	0.07	0.917
MVPA daily time (min)	**10**	**31.8**	**15.4**	**10**	**58.6**	**17.7**	**0.004**	**5**	**45.2**	**8.91**	**5**	**73.0**	**8.55**	**0.009**	**5**	**18.4**	**1.22**	**5**	**44.1**	**10.6**	**0.009**
Total cholesterol (mg/dL)	10	140	23.5	9	167	34.1	0.060	5	132	16.3	5	163	34.4	0.117	5	148	28.6	4	171	38.4	0.327
HDL-c (mg/dL)	10	50.0	12.0	9	52.6	12.7	0.623	5	54.8	15.3	5	56.2	13.2	0.834	5	45.2	5.89	4	48.0	12.1	0.805
LDL-c (mg/dL)	10	78.7	24.5	9	103	25.2	0.060	5	68.6	24.1	5	96.7	23.1	0.175	5	88.8	22.6	4	111	28.7	0.142
Non-HDL-cholesterol (mg/dL)	10	90.4	26.4	9	114	26.5	0.102	5	77.6	24.1	5	107	22.1	0.117	5	103	24.2	4	123	31.9	0.219
Total cholesterol/HDL	10	2.94	0.72	9	3.24	0.65	0.775	5	2.60	0.86	5	2.93	0.27	0.465	5	3.28	0.37	4	3.64	0.81	0.712
Triacylglycerides (mg/dL)	10	58.6	22.3	9	54.4	21.4	0.488	5	45.0	0.00	5	49.4	9.84	0.317	5	72.2	25.7	4	60.8	31.5	0.371
Glucose (mg/dL)	10	81.7	7.41	9	86.3	9.79	0.253	5	82.8	9.68	5	81.4	6.54	1.000	5	80.6	5.18	4	92.5	10.3	0.050
Insulin (µIU/mL)	9	6.15	4.83	7	5.87	3.53	0.791	5	5.44	2.36	3	5.90	4.58	0.655	4	7.05	7.28	4	5.85	3.31	0.773
HOMA-IR	9	1.25	0.97	7	1.29	0.77	0.560	5	1.15	0.56	3	1.23	0.99	0.655	4	1.39	1.42	4	1.34	0.73	0.564
HbA1c [%]	9	5.43	0.18	9	5.19	0.40	0.226	5	5.44	0.21	5	5.20	0.52	0.915	4	5.42	0.17	4	5.18	0.26	0.144
hsCRP (mg/dL)	9	0.27	0.29	8	0.18	0.24	0.664	5	0.17	0.18	4	0.26	0.34	0.806	4	0.39	0.38	4	0.11	0.03	0.245

Statistically significant differences between children with low and high MVPA level were assessed by *U* the Mann–Whitney and are indicated in bold. *BMI* body mass index, BMI Z-score, according to Cole et al.^32^, *FFMI* Fat-free mass index, *MVPA* moderate-vigorous physical activity, *HDL-Cholesterol* high-density lipoprotein cholesterol, *LDL-Cholesterol* low-density lipoprotein cholesterol, *HOMA-IR* homeostasis model assessment for insulin resistance, *HbA1c* glycated hemoglobin, *hsCRP* high sensitivity C-reactive protein.

### Gene expression results

A total of 45,220 probes were included in the microarray analysis. Of them, 29,148 probes remained after background adjustment, normalization, and merge of replicated probes, and were considered for further analyses. In total, 166 genes were found to be differentially expressed in children with low vs. high (the reference group) MVPA values (p < 0.01, Limma test), although none was altered after FDR adjustment.

**Figure 2:**
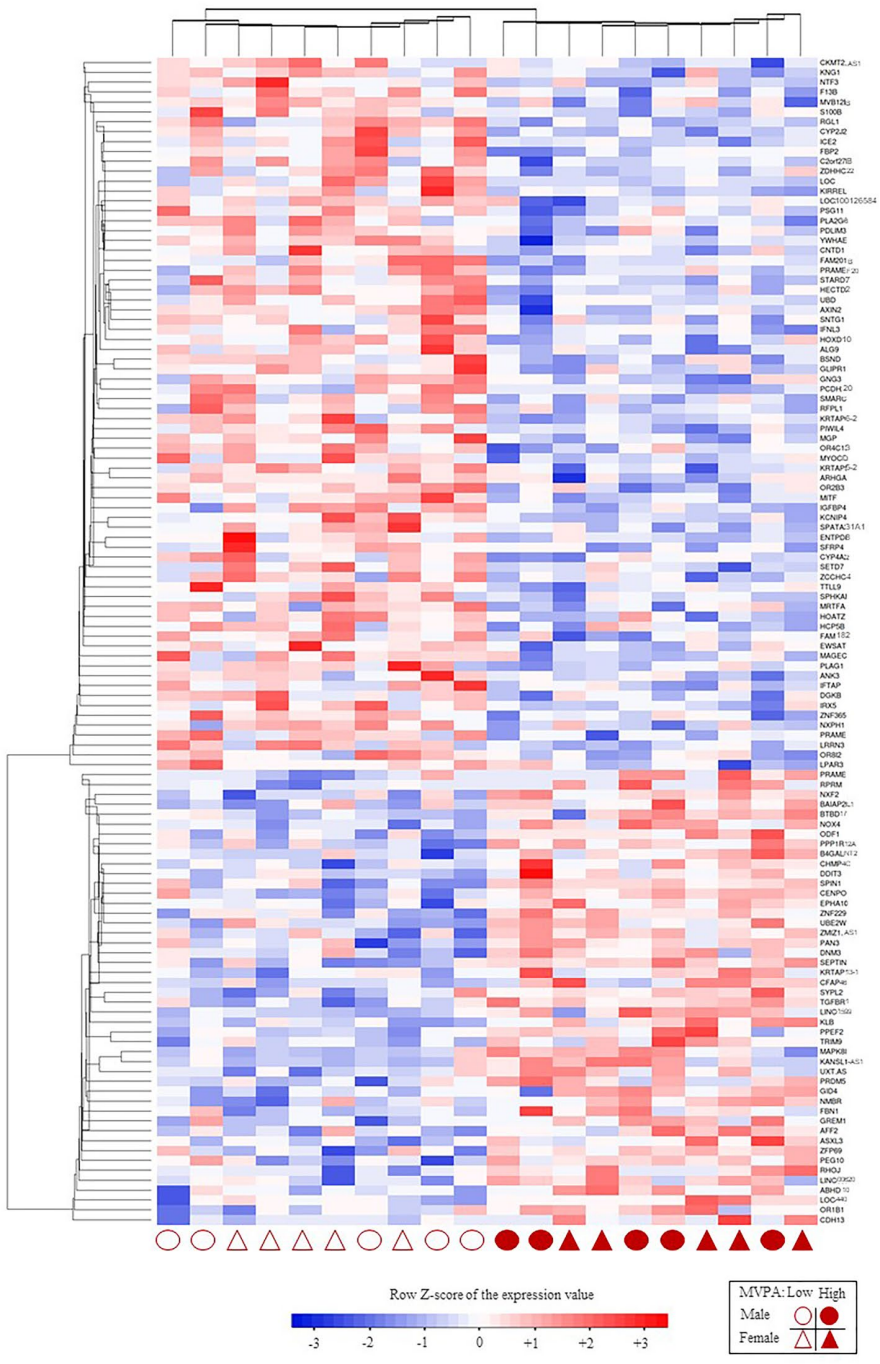
Heatmap representing individual expression data of genes differentially expressed in PBC between children with low and high MVPA levels from the IDEFICS study Spanish cohort. Rows represent the set of 118 differentially expressed genes with known function. Hierarchical centroid linkage (with Pearson distance) was the clustering method selected. Columns represent log2-transformed gene expression values for each gene in every single subject. Column (individuals) and row (gene expression) values were sorted by centroid linkage clustering method. The Heatmap has been generated with the "Heatmapper tool" (http://www2.heatmapper.ca/expression/)^30^.

**Figure 3:**
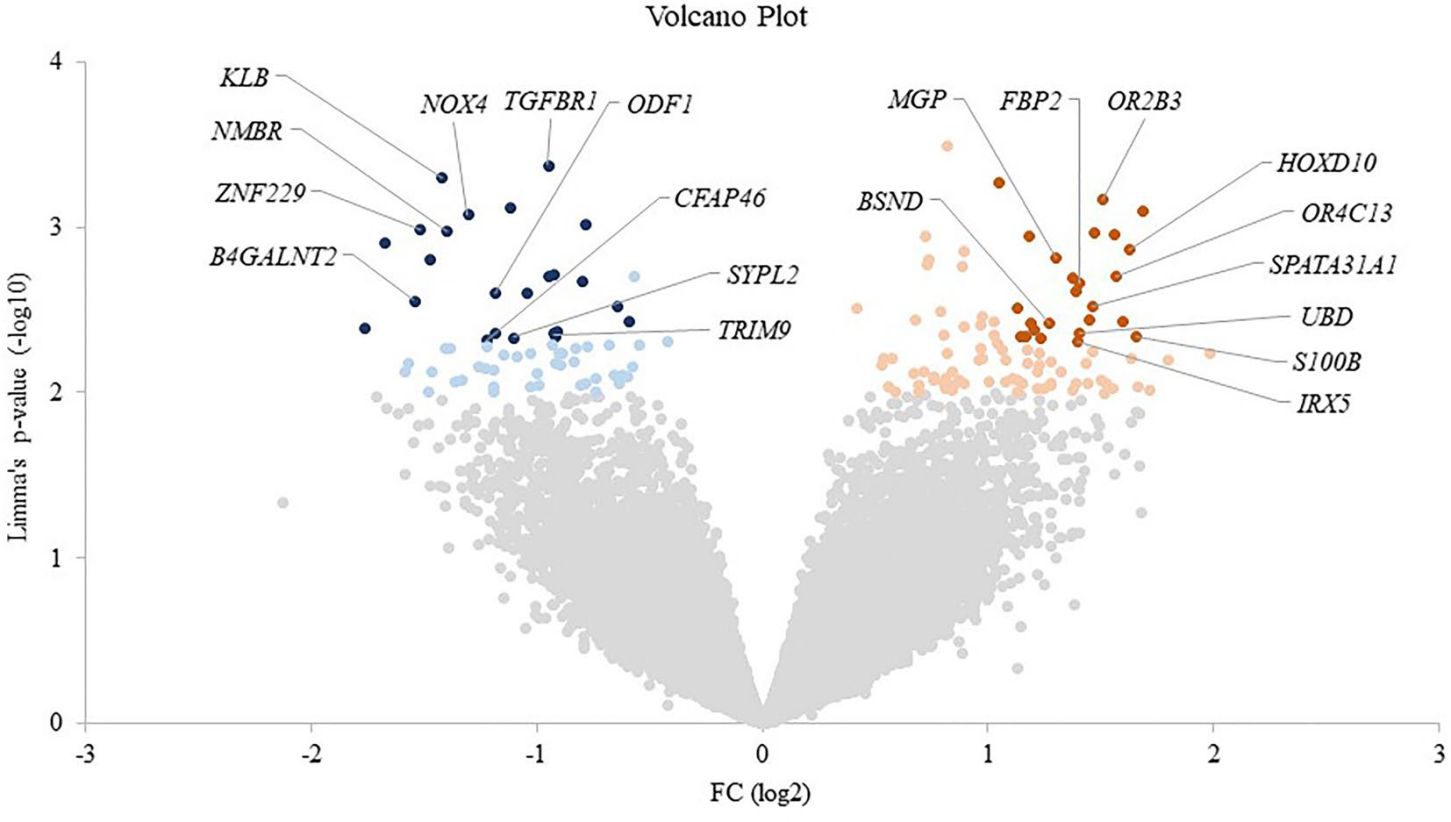
Volcano Plot for all probes included in PBC microarray analysis after background correction, normalization and merging of the replicated probes. The negative log10 of Limma’s test p-value for each probe are plotted against the difference (expressed as fold change, FC, log10 value) between children with low vs. high MVPA levels. Genes with p-value < 0.01 with positive FC (up-regulated in low MVPA children vs. high) are represented in orange and with negative FC (down-regulated in low MVPA children vs. high) are in blue. In addition, the 25 up- and down-regulated genes with p-value < 0.005 appear in darker color. The names of the top-10 up- and down-regulated genes with known function are also indicated.

After searching for the biological function of the differentially expressed gene set, a total number of 118 were genes with a known function (including 72 up-regulated and 46 down-regulated genes), and the remaining 48 genes were classified as unknown (44) or as pseudogenes (4) (Supplementary Fig. 1). A heatmap showing individual gene expression of the 118 genes with known function was carried out for a clearer visualization of the differential expression between subjects with low vs. high MVPA level (Fig. 2). In fact, the two main clusters perfectly distinguish both groups of children, without a separation according to sex in each group of PA. The Volcano Plot representing gene expression in both groups of children (in terms of log2 value of the Fold Change (FC) and the significance value, expressed as log10 of the Limma’s test p-value) shows the genes with significantly higher (positive FC) and lower (negative FC) expression in less active children (Fig. 3). The top-10 down- and up-regulated genes (p < 0.005) with known function are also indicated.

### Functional and biological analysis

**Figure 4:**
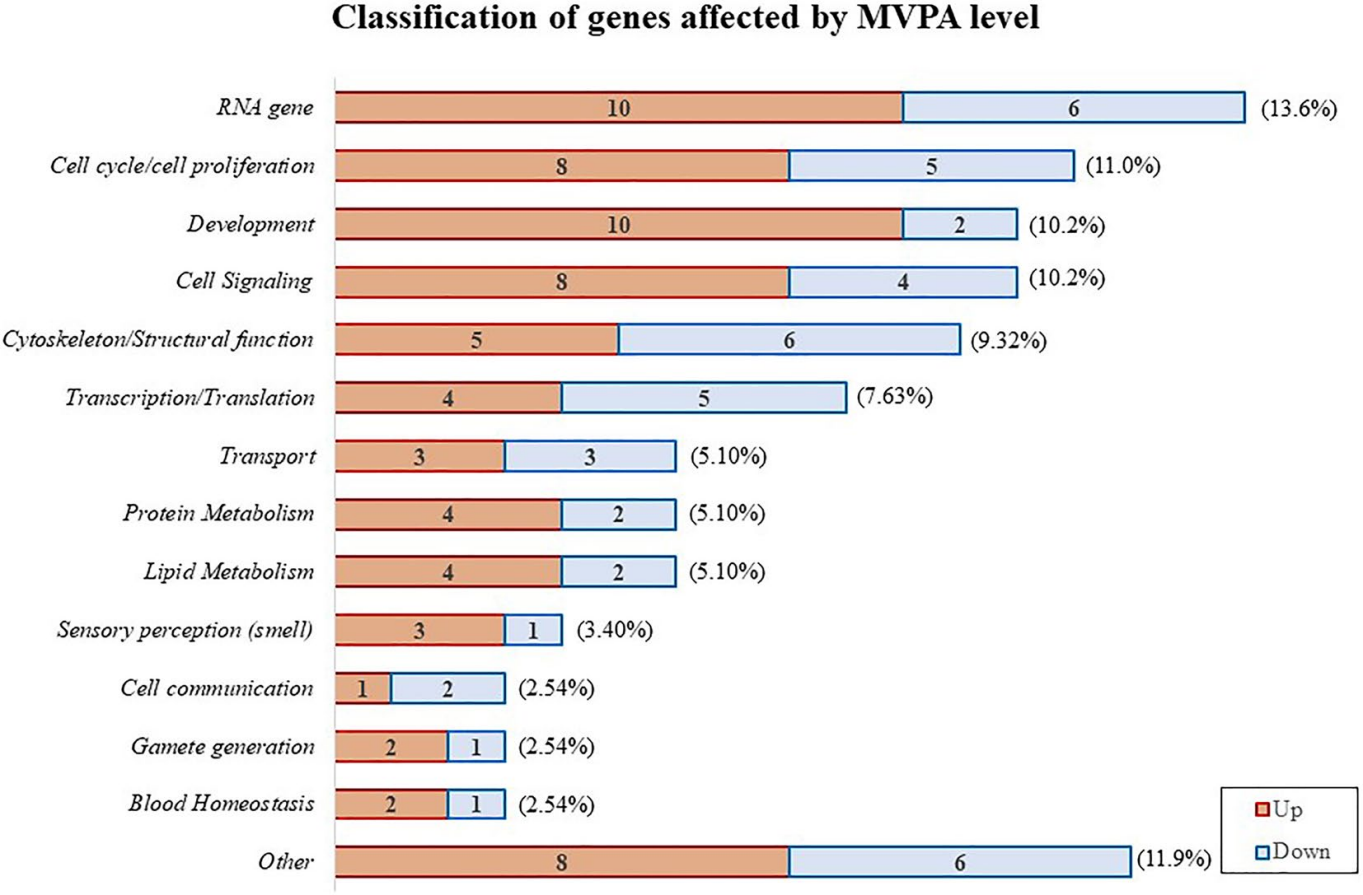
Classification of the 118 known genes differently expressed (p < 0.01) depending on children’s MVPA levels (low or high), into biological processes according to their main biological function. The numbers of up- (red) or down- (blue) regulated genes in children with lower physical activity versus those with higher activity are also indicated. The percentage of genes in each biological function vs. total differentially expressed are shown in parentheses.

The 118 genes with a known function and differentially expressed in children with low compared to high MVPA levels were classified into different biological processes according to the information available in databases (Genecards and NCBI) (Fig. 4). Of them, 16 genes were classified as *RNA genes*, being the group with the highest number of differentially expressed genes. Other biological processes more represented were those related to the *cell cycle and proliferation* (n = 13), *cell signaling* (n = 12), *development* (n = 12), *cytoskeleton or structural function* (n = 11), the *transcription or translation machinery* (n = 9), *cellular transport* (n = 6), and *lipid* (n = 6) and *protein* (n = 6) *metabolism*. Other biological processes with a relevant number of differently expressed genes (n = 3–5) were related to the *sensory perception of smell*, *blood homeostasis*, *gamete formation*, and *cell–cell communication*. Moreover, genes related to biological processes such as *epigenetics*, *immune system*, *vision perception*, *redox balance management* as well as *nucleotide and carbohydrate metabolism* (with < 3 genes involved in each process) were grouped into "others".

### Top ten up- and down-regulated genes

The 20 genes with the highest difference in their expression between groups, including top ten with up-regulated and the same number of down-regulated in children with lower PA in comparison with those with higher PA, with a significance level of p < 0.005 and a characterized biological function in the databases, are indicated in Fig. 3 and more information about them is presented in Table 3 and Supplementary Table 1. On the one hand, considering the ten most down-regulated genes, three of them are involved in cell signaling procedures (*KLB*, FC = − 1.42; *NMBR*, − 1.40 and *TGFBR1*, − 0.95) and the other seven have a role in different biological processes, such as blood homeostasis (*B4GALNT2*, − 1.54), transcription (*ZNF229*, − 1.52); redox metabolism (*NOX4*, − 1.31), cytoskeleton/structural function (*CFAP46*, − 1.22), gamete generation (*ODF1*, − 1.18), transport processes (*SYPL2*, − 1.10), and protein metabolism (*TRIM9*, − 0.92). On the other hand, among the ten most up-regulated genes, three are involved in developmental processes (*S100B*, + 1.66; *HOXD10*, + 1.62; *IRX5*, + 1.40), two in olfactory perception (*OR4C13*, + 1.57; *OR2B3*, + 1.51), and the remaining five genes are related with gamete generation (*SPATA31A1*, + 1.46), protein (*UBD*, + 1.41) and carbohydrates (*FBP2*, + 1.41) metabolism, cytoskeleton/structural function (*MGP*, + 1.30), and the ion channel transport (*BSND*, + 1.27).

**Table 3:** Top 10 down- and up-regulated most significant (p < 0.005) genes by physical activity level in PBC ranked by Fold Change (FC, log2) indicating up-regulation (+) and down-regulation (−) by low physical activity, the Limma's p-value and FDR adjusted p-value.

Gene symbol	Gene name	Sequence ID	Biological process	FC	p-value	p (adj.)
*B4GALNT2*	Beta-1,4-N-acetyl-galactosaminyltransferase 2	NM_153446	*Blood homeostasis*	− 1.54	2.77E−03	0.999968
*ZNF229*	Zinc finger protein 229	NM_014518	*Transcription/translation*	− 1.52	1.02E−03	0.999968
*KLB*	Klotho beta	NM_175737	*Cell signaling*	− 1.42	4.94E−04	0.999968
*NMBR*	Neuromedin B receptor	NM_002511	*Cell signaling*	− 1.40	1.05E−03	0.999968
*NOX4*	NADPH oxidase 4	NM_016931	*Redox Metabolism*	− 1.31	8.25E−04	0.999968
*CFAP46*	Cilia and flagella associated protein 46	NM_173572	*Cytoskeleton/structural function*	− 1.22	4.75E−03	0.999968
*ODF1*	Outer dense fiber of sperm tails 1	NM_024410	*Gamete generation*	− 1.18	2.50E−03	0.999968
*SYPL2*	Synaptophysin like 2	NM_001040709	*Transport*	− 1.10	4.63E−03	0.999968
*TGFBR1*	Transforming growth factor beta receptor 1	NM_004612	*Cell signaling*	− 0.95	4.17E−04	0.999968
*TRIM9*	Tripartite motif containing 9	NM_015163	*Protein metabolism*	− 0.92	4.51E−03	0.999968
*BSND*	Barttin CLCNK type accessory beta subunit	NM_057176	*Transport*	+ 1.27	3.82E−03	0.999968
*MGP*	Matrix Gla protein	NM_001190839	*Cytoskeleton/structural function*	+ 1.30	1.54E−03	0.999968
*IRX5*	Iroquois homeobox 5	NM_005853	*Development*	+ 1.40	4.89E−03	0.999968
*FBP2*	Fructose-bisphosphatase 2	NM_003837	*Carbohydrate metabolism*	+ 1.41	2.19E−03	0.999968
*UBD*	Ubiquitin D	NM_006398	*Protein metabolism*	+ 1.41	4.34E−03	0.999968
*SPATA31A1*	SPATA31 subfamily A member 1	NM_001040065	*Gamete generation*	+ 1.46	3.01E−03	0.999968
*OR2B3*	Olfactory receptor family 2 subfamily B member 3	NM_001005226	*Sensory perception (smell)*	+ 1.51	6.69E−04	0.999968
*OR4C13*	Olfactory receptor family 4 subfamily C member 13	NM_001001955	*Sensory perception (smell)*	+ 1.57	1.98E−03	0.999968
*HOXD10*	Homeobox D10	NM_002148	*Development*	+ 1.62	1.36E−03	0.999968
*S100B*	S100 calcium binding protein B	NM_006272	*Development*	+ 1.66	4.57E−03	0.999968

Genes are ranked by FC.

### Analysis of most affected biological pathways

Beyond the manual classification of the main function of genes, the complete set of genes with differential expression (p-value < 0.01) between subjects with lower vs. higher MVPA levels was also computationally classified using the *Metascape* database^31^. According to *Metascape* assay, 41 genes were excluded from the analysis because they were considered unknown genes. Therefore, the remaining 125 genes were classified according to their involvement in biological clusters. The results of the clustering of the genes affected by the level of PA in the top ten biological pathways are shown in Table 4. In that context, the most significantly enriched biological pathways were related with cell signaling and communication processes, such as “*positive regulator of receptor internalization*”, “*regulation of transmembrane receptor protein serine/threonine kinase signaling pathway*”; “*G alpha (q) signaling events*”; “n*egative regulation of protein phosphorylation*”. In addition, other pathways were related to the organism development and growth procedures, such as the “*skeletal system morphogenesis*”; “*cell projection assembly*”; “*gamete generation*”; and “*cytoskeleton-dependent cytokinesis*”. It is remarkable that the up-regulation of a core of genes was included in more than one of the aforementioned biological pathways, including *DKGB, HOATZ, KNG1, LPAR3, MYOCD, NTF3*, and *SFRP4*. Especially noteworthy is the case of *SFRP4*, as this gene is involved in 3 of the 10 pathways most affected by the MVPA duration (Table 4).

**Table 4:** Detailed classification of the top 10 enriched pathways most affected by MVPA levels, with p-value, q-value (multi-test adjusted p-value), ratio and up- and down-regulated genes, according with Meta Scape clustering.

#	Most associated Pathway	p-value (log)	q-value (log)	Ratio	Up-regulated genes	Down-regulated genes
1	*Positive regulation of receptor internalization*	− 3.73	0	3/27	*NTF3*; *SFRP4*	*GREM1*
2	*Skeletal system morphogenesis*	− 3.45	0	6/225	*HOXD10*; *MGP*; *SFRP4*; *AXIN2*; *IRX5*	*TGFBR1*
3	*Regulation of transmembrane receptor protein serine/threonine kinase signaling pathway*	− 3.06	0	6/267	*SFRP4*; *MYOCD*	*FBN1*; *TGFBR1*; *PEG10*; *GREM1*
4	*Cell projection assembly*	− 2.76	0	7/415	*LPAR3*; *HOATZ*	*CDH13*; *SEPTIN2*; *DNM3*; *CFAP46*; *RHOJ*
5	*G alpha (q) signaling events*	− 2.68	0	5/216	*DGKB*; *GNG3*; *KNG1*; *LPAR3*	*NMBR*
6	*Protein catabolic process*	− 2.62	0	9/693	*UBD*; *MAGEC2*; *MVB12B*	*DDIT3*; *UBE2W*; *ABHD10*; *GID4*; *CHMP4C*; *TRIM9*
7	*Wound healing*	− 2.62	0	6/326	*DGKB*; *F13B*; *KNG1*; *MRTFA*	*TGFBR1*; *CHMP4C*
8	*Negative regulation of protein phosphorylation*	− 2.52	0	6/341	*NTF3*; *KIRREL2*; *MYOCD*	*PPEF2*; *MAPK8IP1*; *GREM1*
9	*Gamete generation*	− 2.49	0	9/725	*IFTAP*; *CNTD1*; *PIWIL4*; *HOATZ*; *SPATA31A1*	*SEPTIN2*; *ODF1*; *TGFBR1*; *SPIN1*
10	*Cytoskeleton-dependent cytokinesis*	− 2.14	0	3/95	*ANK3*	*SEPTIN2*; *CHMP4C*

Likewise, up to four down-regulated genes were present in different biological pathways (*CHMP4C, GREM1, SEPTIN2*, and *TGFBR1*). For instance, *TGFBR1* is involved in four biological pathways. On the other hand, a significant number of differentially expressed genes in children with a lower vs. higher level of PA were classified in “*wound healing*” and in “*protein catabolic processes*”. In relation to those involved in protein catabolism, a greater number of genes were down-regulated (6) in children with a low PA compared to those up-regulated (3). In contrast, a reverse pattern was observed for the *wound healing processes*, with four up-regulated genes and two down-regulated.

## Discussion

We used the results of a whole-genome transcriptomic analysis, previously carried out in PBC from children with and without overweight/obesity^19^, to identify the genes differently expressed between subjects with shorter and longer accelerometer-measured MVPA duration (min/day). In comparison to PA questionnaires, which are considered imprecise in the assessment of PA, accelerometers provide more objective measures of PA, and have been considered the most valid method of assessing PA in children under free-living conditions^33,34^. As previously described, boys displayed longer daily MVPA durations compared to girls^34^ and only boys with MVPA duration over the median value complied with the stablished recommendations of 60 or more minutes of MVPA per day^35^.

The comparison between children with shorter MVPA durations vs. those with longer durations showed no significant differences regarding anthropometric characteristics or biochemical markers of cardiometabolic status, at this early age. However, the gene expression analysis in blood cells allowed the identification of 166 genes differentially expressed between both groups. Among them, a total of 118 were genes with a known function (72 of them were up-regulated and 46 down-regulated in children with lower PA in comparison to children with higher PA), so both groups were clearly distinguished in the heatmap.

The top ten genes with a known function that were down-regulated were mainly related to cell signaling (*KLB*, *NMBR*, *TGFBR1*), transcription machinery (*ZNF229*), blood homeostasis (*B4GALNT2*), redox metabolism (*NOX4*), structural function or involved in cytoskeleton (*CFAP46*), gamete generation (*ODF1*), transport processes (*SYPL2*) and protein metabolism (*TRIM9*), whereas the top ten known genes up-regulated in individuals with low PA were related to development (*S100B*, *IRX5*, *HOXD10*), olfactory perception (*OR2B3*, *OR4C13*), carbohydrate metabolism (*FBP2*), cellular management protein (*UBD*), gamete generation (*SPATA31A1*), extracellular matrix (*MGP*), and transport (*BSND*). Information on the proteins encoded by these genes and a summary on their potential functions are included as supplementary material (Supplementary Table 1).

The down-regulation derived from low PA levels for some of the identified genes, particularly *KLB* and *NOX4,* is supported by scientific evidence showing their relationship with PA and cardiometabolic health. Concretely, the *KLB* gene encodes for the co-receptor (named Beta Klotho, KLB) of some members of the Fibroblast Growth Factor (FGF) family^36^. Therefore, the contribution of KLB is associated with increased sensitivity to the beneficial effects of FGF21 on obesity and type 2 diabetes, including regulation of body weight, plasma lipid levels and insulin sensitivity^37^. FGF21 signaling in adipose tissues has been highlighted as an important molecular transducer of exercise, exerting beneficial metabolic effects on systemic glucose homeostasis and insulin sensitivity^38^. Interestingly, metabolic alterations of high-fat diet induced-obesity in mice have been related with reduced FGF receptor-1 (*FGFR1*) and *KLB* expression in adipose tissues^38^. In these animals, exercise was shown to induce transcriptional activation of *FGFR1* and *KLB* in adipocytes, resulting in increased sensibility to FGF21, and hence attenuating obesity-induced metabolic dysfunction^38^. Therefore, the presence of decreased *KLB* mRNA levels in PBC of less active children found here may be reflecting the down-regulation occurring in other metabolic tissues and may be considered a candidate early biomarker of the detrimental health effects of this condition. In addition, such changes provide relevant information on molecular mechanisms underlying metabolic alterations that may be associated with insufficient PA, according the WHO recommendations^5^.

Concerning *NOX4*, its protein product (NADPH oxidase 4, NOX4) plays an essential role in cellular adaptations and mitochondrial redox balance in response to exercise^39,40^. For instance, it is involved in the metabolic adaptations in skeletal muscle to chronic exercise, specially promoting fatty acid oxidation^41^. Moreover, NOX4 may also mediate the protective effects of PA against obesity-induced endothelial dysfunction, since *Nox4* deficiency in mice has been related to reduced PA performance and exercise-derived vascular protective outcomes^42^. Moreover, *Nox4* deficiency has been related with reduced maximal exercise capacity and impaired cardiac contractile function during repeated acute physical exercises^39^. Therefore, the activity of both KLB and NOX4 seems to be essential, not only for the preservation of the balanced mitochondrial redox state, but also for the beneficial effects of PA. In this way, the down-regulation of both genes observed in less active children could be indicative of a non-adaptation profile to the conditions of habitual exercise and, consequently, to its potential health benefits.

Besides, *KLB* and *NOX4*, down-regulation of other highlighted genes may also be of interest as potential biomarkers of low PA, because of their relationship with skeletal muscle function. Among them, it is worth mentioning *SYPL2*, which encodes for the Synaptophysin-like protein 2, also known as Mitsugumin 29, a major protein component of the triad junction in skeletal muscle involved in the formation of junctional membrane structures that are important for efficient signal conversion during excitation–contraction coupling. It is involved in normal muscle contraction but may also have a role in muscle fatigue^43^. Specifically, it has been reported that Mitsugumin-29-deficient mice display abnormal skeletal muscle contractility^44^, and both fast-twitch and slow-twitch muscles were more susceptible to fatigue^43^. Changes in the expression of *TGFBR1* and *NMBR* are also worth mentioning, although the biological significance in relation to PA is less clear. *TGFBR1* encodes for the Transforming Growth Factor beta receptor 1 (a serine/threonine protein kinase that transduces the TGF-β signal from the cell surface to the cytoplasm), and physical exercise has been reported to enhance plasma levels of its ligand (TGFB1)^45^, also suggesting a probable stimulation of its receptor. Therefore, the presence of decreased expression levels of *TGFBR1* in less active children would be in accordance with the hypothetical induction by PA performance. Of note, this gene was found to be involved in 4 of the 10 most affected pathways by MVPA, named “Skeletal system morphogenesis”, “regulation of transmembrane receptor protein serine/threonine kinase signaling pathway”, “wound healing”, and “gamete generation”. This may be related to the ability of TGF-β family members to regulate cell proliferation, migration, and differentiation, and therefore they are relevant in many processes related to embryonic development and tissue morphogenesis^46^, tissue damage repair^47^ and reproduction and fertility^48^. The *NMBR* protein product is the G protein-coupled receptor (GPCR) of Neuromedin B. Neuromedin B and its receptor (NMBR) have been recently proposed as regulators of skeletal muscle mitochondrial function^49^, but the significance of *NMBR* down-regulation in PBC of less physically active children remains to be determined.

Unlike the aforementioned genes found to be down-regulated under conditions of low PA, less evidence supports the influence of the PA level on the most significant genes found to be up-regulated under such conditions, despite some of them are involved in relevant biological functions and related with adipose tissue function and inflammation (see Supplementary Table 1). Concretely, *IRX5* is a member of the Iroquois homeobox gene family that is essential for proper vertebrate embryonic development^36^. Interestingly, *IRX5* expression is strongly influenced by regulatory elements located on the fat mass and obesity-associated (*FTO*) gene sequence^50^, which has been reported as one of the strongest genetic predictors of obesity in genome-wide association studies^51,52^. In this sense, the presence of genetic variants in *FTO* associated with increased risk of obesity is also influencing higher *IRX5* mRNA levels in adipocytes, causing decreased energy expenditure and increased lipid accumulation^50^. Therefore, increased *IRX5* expression as shown in PBC of children with low PA could be indicative of an altered adipocyte function and related with obesity predisposition. *UBD* gene encodes for ubiquitin D, a member of the ubiquitin-like modifier family, which is mainly expressed in immune system cells but also in other cell types, and its expression is highly inducible by pro-inflammatory cytokines^53^. Thus, there is a clear relationship between overexpression of this gene and inflammation. Expression levels of *Ubd* were found to be decreased in skeletal muscle arterioles of rats that underwent exercise training programs^54^ what seems to be consistent with our findings showing higher expression levels in PBC of less active children.

*MGP* encodes for a member of vitamin K2 dependent protein named Matrix Gla Protein (MGP) that acts as an inhibitor of vascular mineralization and participates in bone organization^55^. In addition, MGP has been described to be involved in fat metabolism. It is highly expressed in mice adipose tissues, especially in visceral fat, and high levels in serum of inactive MGP have been proposed as a marker for central adiposity^56^. Although the direct effect of PA on MGP expression has not been previously assessed, present results suggest that PA may modulate the expression of this gene in PBC, but this association needs to be further assessed.

The enrichment analysis of functional biological pathways showed that one of the most affected pathways comparing lower vs. higher PA level was Protein catabolism, with higher presence of down-regulated genes in children with lower PA (*DDIT3*, *UBE2W*, *ABHD10*, *GID4*, *CHMP4C*, and *TRIM9*) with respect to those up-regulated (*UBD*, *MAGEC2*, and *MVB12B*). Among other pathways affected, it is worth mentioning that of Skeletal system morphogenesis, which could be supported by the impact of exercise on the expression of bone morphogenetic proteins, associated with the prevention of progression of bone lesions observed in an injured animal model^57^. The association among PA, sedentary behavior and bone stiffness has been previously assessed in European children and adolescents, from the IDEFICS study^58^. In that context, sedentary behavior was associated with a higher risk of developing alterations in bone growth, especially in overweight/obese subjects^58^. Hence, the changes observed in gene expression involved in skeletal system morphogenesis could be indicative of potential bone development disorders derived from low PA performance. Another top affected pathway by low PA level was associated with Wound healing processes. Physical training has been shown to accelerate wound healing in both mice and humans compared to sedentary behaviors^59^. Therefore, the transcriptomic modifications observed in genes related with this pathway could indicate the modulation of wound repair processes depending on the degree of PA.

Despite clearly different expression profiles observed between less physically active children compared to the more active ones, the study has some limitations. First, it is worth noting the limited number of individuals finally used for gene expression comparison analysis (n = 20), despite the well compensation of sex and age ranges between groups. Second, changes on potential transcript-based biomarkers by sedentary behaviours here analysed in "free-living” children were expected to be small. Consequently, although multiple testing restrictive corrections (FDR) were assessed, the unadjusted p-values were considered to identify the differences in transcript levels. However, a more restrictive significance threshold (p < 0.01), rather than the conventional (p < 0.05), and the Limma test (which provides a substantial improvement in terms of false positives than t-test, especially for small samples^60^) were used to reduce type I error in microarray data analyses. Third, the cross-sectional design of the study can also be considered a technical limitation since it cannot go further in providing definitive information on the observed cause-effect relationships. Furthermore, potential confounding factors not included in analyses (e.g., children’s dietary habits) or non-reported/unknown (such as endocrine disruptors exposures^61^), could have an impact on gene expression and it may lead to limitations in interpreting results that may be taken into account in future studies. Therefore, more studies are needed to validate the predictive use of the biomarkers identified here.

In conclusion, functional blood cell microarray analysis in less physically active children compared to more physically active children has shown early differences in gene expression profile associated with the levels of PA. The most notable changes observed associated to low PA have been the down-regulation of genes related to cardiometabolic benefits (*KLB*, *NOX4)* and improved skeletal muscle function (*SYPL2*), together with the up-regulation of genes associated with increased fat accumulation and metabolic complications (*IRX5*, *UBD* and *MGP*). Therefore, considering that subjects with a low level of PA did not show anthropometric and biochemical differences compared to those with a high level, expression levels of the aforementioned genes in PBC, if validated in future studies, could be of interest as candidate biomarkers of increased risk for future cardiometabolic problems associated to physical inactivity, even before changes in classical biochemical markers are observable. In addition, the pathway enrichment analysis revealed potential alterations in protein catabolism, skeletal system morphogenesis, and wound healing processes associated to low PA. Thus, gene expression analysis in PBC represents a useful approach for the early identification of alterations in metabolic processes and pathways associated to insufficient PA and may help in monitoring their potential reversion by personalized interventions.

## Supplementary Information

Supplementary Figure 1.

Supplementary Table 1.

## Data Availability

The datasets generated during and/or analysed during the current study are available from the corresponding author on reasonable request.
