# Supplementary material for: KLB and NOX4 expression levels as potential blood-based transcriptional biomarkers of physical activity in children
Source: Sci Rep. 2023 Apr 5;13:5563. doi: 10.1038/s41598-023-31537-4 (PMC10074339; doi:10.1038/s41598-023-31537-4)
Supplement: Supplementary file 1 — Supplementary Figure 1. [file 41598_2023_31537_MOESM1_ESM.pdf]

Supplementary Figure 1

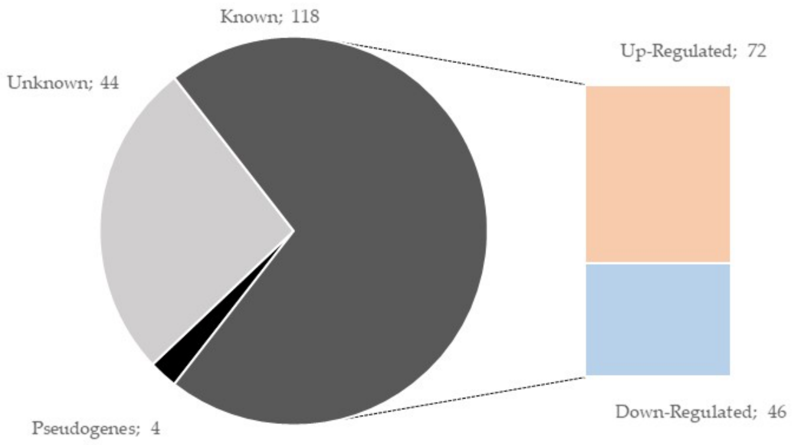

Pie chart indicating the number of unknown genes, pseudogenes, and known genes up and down-regulated after manual classification of genes with differential expression in Low vs. High MVPA children
