# Supplementary material for: KLB and NOX4 expression levels as potential blood-based transcriptional biomarkers of physical activity in children
Source: Sci Rep. 2023 Apr 5;13:5563. doi: 10.1038/s41598-023-31537-4 (PMC10074339; doi:10.1038/s41598-023-31537-4)
Supplement: Supplementary file 2 — Supplementary Table 1. [file 41598_2023_31537_MOESM2_ESM.pdf]

Supplementary Table 1. Function of the known genes, which expression was most affected (top 10 down-, and up-regulated) by low physical activity level. Genes are arranged in alphabetical order.

**Gene symbol**	**Gene name**	**Encoded protein**	**Function**
B4GALNT2	Beta-1,4-N-acetyl-galactosaminyltransferase 2	Beta-1,4-N-acetyl-galactosaminyltransferase 2	Enzyme involved in both Sd(A) and Cad antigens biosynthesis. It catalyzes the last steps of each antigen biosynthesis [1] and its activity has been detected in different body fluids, including plasma and urine [2].
BSND	Barttin CLCNK type accessory beta subunit	Barttin	Key subunit of a channel crucial for sodium chloride reabsorption. *In vivo* evidence suggest an important role in blood pressure regulation [3].
CFAP46	Cilia and flagella associated protein 46	Cilia- and flagella-associated protein 46	This protein is essential for gamete production and alterations in its function/structure have been associated with the multiple morphological abnormalities of the flagella [4], such as asthenozoospermia. Epigenetic modifications on *CFAP46* gene have been suggested as a potential early biomarker for asthma acquisition during adolescence/young adulthood, in girls [5].
FBP2	Fructose-bisphosphatase 2	Fructose-1,6-bisphosphatase isozyme 2	This enzyme is mainly expressed in muscle and other non-gluconeogenic tissues contributing to muscular glycogen replenishment after exercise in skeletal muscle [6]. *In vitro,* its overexpression in muscle increases gluconeogenic flux and glucose uptake [7]. It would also play a role in thermogenesis induction, by no yet well-defined mechanisms [6].
HOXD10	Homeobox D10	Homeobox protein Hox-D10	Member of the Abd-B homeobox family with a homeobox DNA-binding domain [8]. In humans, the skeletal muscle *HOXD10* gene results hypermethylated by ageing and the increase in physical activity performance would help to prevent these epigenetic modifications [9]
IRX5	Iroquois homeobox 5	Iroquois-class homeodomain protein IRX-5	Member of the Iroquois family of homeodomain transcription factors that play a role in many developmental processes [8]. A role in obesity has been suggested, since *FTO* genetic variants associated with obesity risk are influencing higher *IRX5* expression [10]. In contrast, *Irx5*-KO mice seem to be protected against obesity development [11].
KLB	Klotho beta	Beta-klotho	Co-receptor of specific members of the Fibroblast Growth Factor (FGF) family, acting as an enhancer of the ability to bind FGF21 [8]. Therefore, KLB plays a role in the cell sensitivity to FGF21 effects [12].
MGP	Matrix Gla protein	Matrix Gla protein	Member of vitamin K2 dependent protein that acts as an inhibitor of vascular mineralization and participates in bone organization [13].

			Elevated plasma levels of inactive MGP have been suggested as a potential bio-markers of central adiposity [14].
NMBR	Neuromedin B receptor	Neuromedin-B receptor	G protein-coupled receptor of Neuromedin B, a bombesin-related peptide involved in signaling pathways, such as muscle contraction or blood pressure and glucose homeostasis [15]. *NMBR* is expressed in cell surface of white adipocytes and *in vitro* inhibition by antagonism inhibits early pre-adipocyte differentiation [16].
NOX4	NADPH oxidase 4	NADPH oxidase 4	It plays a critical role in cellular adaptations and mitochondrial redox equilibrium in response to exercise [17,18], such as fatty acid oxidation induction, in skeletal muscle [19]. The activity of this protein as a dissipator of free radicals would be related to beneficial effects derived from physical activity performance [20]. Moreover, the lack of this protein in animal models is related to decreased maximum capacity to perform acute and repeated physical exercises as well as with alterations in cardiac muscle contraction [17].
ODF1	Outer dense fiber of sperm tails 1	Outer dense fiber protein 1	ODF1 is the major protein of the outer fiber of the mammalian sperm tail and is functionally involved in sperm differentiation and morphogenesis [21]. ODF1 partial deficiency in male mice results in reduced sperm motility [22].
OR2B3	Olfactory receptor family 2 subfamily B member 3	Olfactory receptor 2B3	Member of a large family of olfactory receptors, structurally classified as G protein-coupled receptors, that initiate neuronal responses when interact with odorant particles and, consequently trigger smell perception [23]. Genetic variants in *OR2B3* are associated with type 1 diabetes mellitus [24].
OR4C13	Olfactory receptor family 4 subfamily C member 13	Olfactory receptor 4C13	OR4C13 is an olfactory receptor member involved in smell perception [23]. It is expressed on sperm cell surface, and it may have a role in its maturation and migration [25].
S100B	S100 calcium binding protein B	Protein S100-B	Member of a multigenic family of small Ca2+-binding proteins of the EF-hand type highly abundant in brain, but also it is widely expressed in adipocytes and skeletal muscle [26]. This protein has a role in cell development, but under conditions of cell damage or necrosis, S100B is passively released outside the cell [27].
SPATA31A1	SPATA31 subfamily A member 1	Spermatogenesis-associated protein 31A1	SPATA31A1 is predicted to be involved in cell differentiation and spermatogenesis [8]. Despite *SPATA31* genes products are implicated in detection and repairing UV-induced DNA damage, *in vitro* over-

			expression of *SPATA31A1* leads to premature senescence in primary human fibroblasts [28].
SYPL2	Synaptophysin like 2	Synaptophysin-like protein 2	SYPL2 is the major protein component of the triadic junction in the skeletal muscle involved in the development of membrane junction structures, playing an essential role for muscle contractibility [29]. Mice lacking this gene present abnormal skeletal muscle contractility [30]. In addition, this protein is also expressed in brain, where a role in food intake regulation, affecting the central reward systems, is hypothesized [31]. Thus, the presence of a low frequency genetic variant (rs62623713) in *SYPL2* is associated with morbid obesity in humans [32].
TGFBR1	Transforming growth factor beta receptor 1	TGF-beta receptor type-1	It is a transmembrane serine/threonine kinase receptor involved in the signal transduction of some members of the transforming growth factor beta (TGFB) family, from the cell surface to the cytoplasm. Thus, this receptor is essential in modulating the regulation of various physiological and pathological processes, including cell cycle control, cell differentiation, wound healing, extracellular matrix production, or immunosuppression [33].
TRIM9	Tripartite motif containing 9	E3 ubiquitin-protein ligase TRIM9	Member of the tripartite motif containing family of E3 ubiquitin ligases, implicated in biological procedures such as inflammation modulation, probably via regulation of nuclear factor-kappa B activity [34]
UBD	Ubiquitin D	Ubiquitin D	Member of the ubiquitin-like modifier family primarily expressed in cells of the immune system, but highly inducible in other cell types by pro-inflammatory cytokines [35]. E.g., its expression is induced by interleukine-1β and interferon-γ in rodent and human pancreatic beta cells [36].
ZNF229	Zinc finger protein 229	Zinc finger protein 229	*ZNF229* is predicted to be a potential regulator of RNA polymerase II activity [8], but there is no direct evidence of its implication in cardiometabolic health so far.

References

1. Montiel, M.-D., Krzewinski-Recchi, M.-A., Delannoy, P. & Harduin-Lepers, A. Molecular cloning, gene organization and expression of the human UDP-GalNAc:Neu5Acalpha2-3Galbeta-R beta1,4-N-acetylgalactosaminyltransferase responsible for the biosynthesis of the blood group Sda/Cad antigen: evidence for an unusual extended cytoplasmic. *Biochem. J.* 373, 369–79 (2003).
2. Takeya, A., Hosomi, O. & Kogure, T. Identification and characterization of UDP-GalNAc: NeuAc alpha 2-3Gal beta 1-4Glc(NAc) beta 1-4(GalNAc to Gal)N-acetylgalactosaminyltransferase in human blood plasma. *J. Biochem.* 101, 251–9 (1987).
3. Nomura, N. *et al.* Role of ClC-K and barttin in low potassium-induced sodium chloride cotransporter activation and hypertension in mouse kidney. *Biosci. Rep.* 38, (2018).
4. Cassuto, N. G. *et al.* Molecular Profiling of Spermatozoa Reveals Correlations between Morphology and Gene Expression: A Novel Biomarker Panel for Male Infertility. *Biomed Res. Int.* 2021, 1–14 (2021).
5. Li, L. *et al.* Newborn DNA methylation and asthma acquisition across adolescence and early adulthood. *Clin. Exp. Allergy* (2022) doi:10.1111/cea.14091.
6. Park, H.-J. *et al.* The essential role of fructose-1,6-bisphosphatase 2 enzyme in thermal homeostasis upon cold stress. *Exp. Mol. Med.* 52, 485–496 (2020).
7. Bakshi, I. *et al.* Fructose bisphosphatase 2 overexpression increases glucose uptake in skeletal muscle. *J. Endocrinol.* 237, 101–111 (2018).
8. Safran, M. *et al.* The GeneCards Suite. *Pract. Guid. to Life Sci. Databases* 27–56 (2021) doi:10.1007/978-981-16-5812-9_2.
9. Turner, D. C. *et al.* DNA methylation across the genome in aged human skeletal muscle tissue and muscle-derived cells: the role of HOX genes and physical activity. *Sci. Rep.* 10, 15360 (2020).
10. Claussnitzer, M. *et al.* FTO Obesity Variant Circuitry and Adipocyte Browning in Humans. *N. Engl. J. Med.* 373, (2015).
11. Bjune, J.-I. *et al.* IRX5 regulates adipocyte amyloid precursor protein and mitochondrial respiration in obesity. *Int. J. Obes.* 43, 2151–2162 (2019).
12. Sonoda, J., Chen, M. Z. & Baruch, A. FGF21-receptor agonists: an emerging therapeutic class for obesity-related diseases. *Horm. Mol. Biol. Clin. Investig.* 30, (2017).
13. Pinto, J. ., Conceição, N., Gavaia, P. . & Cancela, M. . Matrix Gla protein gene expression and protein accumulation colocalize with cartilage distribution during development of the teleost fish Sparus aurata. *Bone* 32, 201–210 (2003).
14. Li, C. *et al.* Matrix Gla protein regulates adipogenesis and is serum marker of visceral adiposity. *Adipocyte* 9, 68–76 (2020).
15. Ohki-Hamazaki, H. Neuromedin B. *Prog. Neurobiol.* 62, 297–312 (2000).
16. de Paula, G. S. M. *et al.* Neuromedin B receptor disruption impairs adipogenesis in mice and 3T3-L1 cells. *J. Mol. Endocrinol.* 63, 93–102 (2019).
17. Hancock, M. *et al.* Myocardial NADPH oxidase-4 regulates the physiological response to acute exercise. *Elife* 7, (2018).
18. Nabeebaccus, A. A. *et al.* The regulation of cardiac intermediary metabolism by NADPH oxidases. *Cardiovasc. Res.* 1–15 (2022) doi:10.1093/cvr/cvac030.
19. Specht, K. S. *et al.* Nox4 mediates skeletal muscle metabolic responses to exercise. *Mol. Metab.* 45, 101160 (2021).
20. Brendel, H. *et al.* NADPH oxidase 4 mediates the protective effects of physical activity against obesity-induced vascular dysfunction. *Cardiovasc. Res.* 116, 1767–1778 (2020).
21. Cabrillana, M. E. *et al.* ODF1, sperm flagelar protein is expressed in kidney collecting ducts of rats. *Heliyon* 5, e02932 (2019).
22. Yang, K. *et al.* The Small Heat Shock Protein ODF1/HSPB10 Is Essential for Tight Linkage of Sperm Head to Tail and Male Fertility in Mice. *Mol. Cell. Biol.* 32, 216–225 (2012).
23. Buck, L. & Axel, R. A novel multigene family may encode odorant receptors: A molecular basis for

odor recognition. *Cell* 65, 175–187 (1991).

24. Qiu, Y., Deng, F., Li, M. & Lei, S. Identification of novel risk genes associated with type 1 diabetes mellitus using a genome-wide gene-based association analysis. *J. Diabetes Investig.* 5, 649–656 (2014).
25. Milardi, D. *et al.* Olfactory Receptors in Semen and in the Male Tract: From Proteome to Proteins. *Front. Endocrinol. (Lausanne).* 8, (2018).
26. Donato, R. S100: a multigenic family of calcium-modulated proteins of the EF-hand type with intracellular and extracellular functional roles. *Int. J. Biochem. Cell Biol.* 33, 637–668 (2001).
27. Sorci, G. *et al.* S100B Protein, a Damage-Associated Molecular Pattern Protein in the Brain and Heart, and Beyond. *Cardiovasc. Psychiatry Neurol.* 2010, 1–13 (2010).
28. Bekpen, C., Xie, C., Nebel, A. & Tautz, D. Involvement of SPATA31 copy number variable genes in human lifespan. *Aging (Albany. NY).* 10, 674–688 (2018).
29. NAGARAJ, R. Y. *et al.* Increased susceptibility to fatigue of slow- and fast-twitch muscles from mice lacking the MG29 gene. *Physiol. Genomics* 4, 43–49 (2000).
30. Nishi, M. *et al.* Abnormal Features in Skeletal Muscle from Mice Lacking Mitsugumin29. *J. Cell Biol.* 147, 1473–1480 (1999).
31. Kurebayashi, N. *et al.* Changes in Ca2+ handling in adult MG29-deficient skeletal muscle. *Biochem. Biophys. Res. Commun.* 310, 1266–1272 (2003).
32. Jiao, H. *et al.* Exome sequencing followed by genotyping suggests SYPL2 as a susceptibility gene for morbid obesity. *Eur. J. Hum. Genet.* 23, 1216–1222 (2015).
33. Wieser, R., Wrana, J. L. & Massagué, J. GS domain mutations that constitutively activate T beta R-I, the downstream signaling component in the TGF-beta receptor complex. *EMBO J.* 14, 2199–2208 (1995).
34. Versteeg, G. A., Benke, S., García-Sastre, A. & Rajsbaum, R. InTRIMsic immunity: Positive and negative regulation of immune signaling by tripartite motif proteins. *Cytokine Growth Factor Rev.* 25, 563–576 (2014).
35. Aichem, A. & Groettrup, M. The ubiquitin-like modifier FAT10 – much more than a proteasome-targeting signal. *J. Cell Sci.* 133, (2020).
36. Brozzi, F. *et al.* Ubiquitin D Regulates IRE1α/c-Jun N-terminal Kinase (JNK) Protein-dependent Apoptosis in Pancreatic Beta Cells. *J. Biol. Chem.* 291, 12040–12056 (2016).
